# Transcriptional profiling of the stringent response mutant strain *E. coli* SR reveals enhanced robustness to large‐scale conditions

**DOI:** 10.1111/1751-7915.13738

**Published:** 2020-12-26

**Authors:** Martin Ziegler, Julia Zieringer, Ralf Takors

**Affiliations:** ^1^ Institute of Biochemical Engineering University of Stuttgart Stuttgart Germany

## Abstract

In large‐scale fed‐batch production processes, microbes are exposed to heterogeneous substrate availability caused by long mixing times. *Escherichia coli*, the most common industrial host for recombinant protein production, reacts by recurring accumulation of the alarmone ppGpp and energetically wasteful transcriptional strategies. Here, we compare the regulatory responses of the stringent response mutant strain *E. coli SR* and its parent strain *E. coli* MG1655 to repeated nutrient starvation in a two‐compartment scale‐down reactor. Our data show that *E. coli* SR can withstand these stress conditions without a ppGpp‐mediated stress response maintaining fully functional ammonium uptake and biomass formation. Furthermore, *E. coli* SR exhibited a substantially reduced short‐term transcriptional response compared to *E. coli* MG1655 (less than half as many differentially expressed genes). *E. coli* SR proceeded adaptation via more general SOS response pathways by initiating negative regulation of transcription, translation and cell division. Our results show that locally induced stress responses propagating through the bioreactor do not result in cyclical induction and repression of genes in *E. coli* SR, but in a reduced and coordinated response, which makes it potentially suitable for large‐scale production processes.

## Graphical abstract

Large‐scale processes suffer from process performance loss. *E. coli* SR exhibits a reduced short‐term transcriptional response when exposed to repeated nitrogen starvation in a scale‐down reactor.

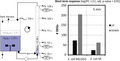

## Introduction

Heterogeneities in large‐scale fed‐batch bioprocesses have long been recognized as a cause for process performance loss at industrial scale compared to homogeneous processes at laboratory scale (Bylund *et al*., 1998). Due to physical, economical and engineering constraints, the generation of gradients in large‐scale reactors is inevitable. Hydrostatic pressure influences the solubility and transfer of gasses, and the mixing time of large reactors can be orders of magnitude higher than that of laboratory reactors producing strong measurable chemical gradients (Larsson *et al*., 1996; Enfors *et al*., 2001; Junker, 2004; Delvigne *et al*., 2006). Common consequences of spatial heterogeneities are loss of productivity, reduced biomass yield, increased byproduct formation and genetic or plasmid instability (Hopkins *et al*., 1987; George *et al*., 1993; Neubauer *et al*., 1995b; Bylund *et al*., 1998; Bylund *et al*., 2000; Jonge *et al*., 2011). Reduced process performance is not limited to a single species but can be observed for many industrial workhorse organisms like *Escherichia coli*, *Saccharomyces cerevisiae, Penicillium chrysogenum* and *Bacillus subtilis* (George *et al*., 1993; Jonge *et al*., 2011; Junne *et al*., 2011; Larsson and Enfors, 1988).

Due to the enormous costs associated with using and maintaining large‐scale equipment, few experiments in the context of academic research have been performed in industrial scale bioreactors (Bylund *et al*., 1999; Bylund *et al*., 2000; Enfors *et al*., 2001). In consequence, researchers have relied on the use of computational fluid dynamics (CFD) to simulate reactor flow fields and on scale‐down reactors to experimentally investigate selected scenarios (Kelly, 2008; Takors, 2012). Various designs of scale‐down reactors exist and have been extensively reviewed elsewhere (Delvigne *et al*., 2017; Delvigne *et al*., 2006; Neubauer and Junne, 2010). One of the commonly used scale‐down reactors follows a multi‐compartment approach: A primary stirred tank reactor (STR) is coupled to a secondary plug flow reactor (PFR). The STR is operated as a well‐mixed compartment under standard limited growth conditions and the PFR simulates a feeding, starvation or anaerobic zone providing the stimulus to be investigated (Lara *et al*., 2006).

Many studies have focused on experimentally simulating the zone close to the feeding point which is usually characterized by substrate excess and potentially oxygen limitation (Enfors *et al*., 2001; Lara *et al*., 2009; Junne *et al*., 2011). For a variety of hosts, common observations in this scenario include the formation of small organic acids and solvents as overflow metabolites or as anaerobic fermentation products (George *et al*., 1993; Neubauer *et al*., 1995b). Ultimately, byproduct formation may lead to process performance loss even if reuptake of byproducts occurs in the well‐mixed limited growth zone (Enfors *et al*., 2001).

Occasionally, starvation zones have attracted attention as well (Neubauer *et al*., 1995a; Neubauer *et al*., 1995b). From CFD simulation and measured data, it is known that distant from the feeding point or close to the reactor walls poorly mixed zones with very low nutrient concentrations exist. An early scale‐down study with *E. coli* employing oscillatory feeding protocols revealed the involvement of the stringent response in the cellular reaction to transient glucose starvation (Neubauer *et al*., 1995a).

The stringent response is a global regulatory program usually preparing *E. coli* for entry into the stationary phase (Magnusson *et al*., 2005; Gaca *et al*., 2015; Hauryliuk *et al*., 2015). Its hallmark is the synthesis of the alarmone (p)ppGpp on short time‐scales by the ribosome‐associated protein RelA or on longer time‐scales by the bifunctional enzyme SpoT (Gallant *et al*., 1970; Atherly, 1979; Murray and Bremer, 1996). ppGpp acts primarily as a transcription factor by binding to RNA polymerase and modulating its affinity to transcription initiation sites and alternative sigma factors. Additionally, ppGpp directly modulates the activity of certain proteins (Dalebroux and Swanson, 2012; Kanjee *et al*., 2011).

The fast and reversible initiation of the stringent response to oscillatory substrate supply was later confirmed by measurements of ppGpp in continuous glucose chemostat cultivations in a two‐compartment stirred tank‐plug flow reactor (STR‐PFR) setup (Löffler *et al*., 2016). The feeding point was placed in the STR creating a starvation zone in the PFR, which allowed to resolve the timescale of cellular response. Moreover, it was shown that extensive transcriptional responses take place as cells move transiently through a nutrient poor zone. From theoretical calculations of ATP costs Löffler *et al*. estimated that an increase in maintenance energy demand of more than 30% was caused by the repeated exposure of cells to the nutrient gradient offering a new explanation for performance losses in large‐scale bioprocesses (Löffler *et al*., 2016). Analogous experiments with ammonium as the limiting nutrient revealed similar, yet less pronounced, regulation patterns affirming the importance of the stringent response for global regulation in *E. coli* in a scenario of oscillating starvation stimuli (Simen *et al*., 2017). Fed‐batch processes limited by ammonium or other nitrogen sources are interesting fermentation scenarios for the production of small molecules which mainly consist of carbon such as fatty alcohols (Chubukov *et al*., 2017). Nitrogen limitation is commonly used to enhance the accumulation of cellular carbon storage products such as polyhydroxyalkanoates used for bioplastic synthesis (Wen *et al*., 2010; Oliveira‐Filho *et al*., 2019), including *E. coli* as a potential host (Wang *et al*., 2009). As nitrogen forms a relatively large part of cells, nitrogen limitation can be easily explored during process development. During scale‐up, such processes will likely suffer from similar issues as carbon‐limited processes (Simen *et al*., 2017).

Recently, the strains *E. coli* SR and *E. coli* HGT with modulated stringent response were constructed in our laboratory (Michalowski *et al*., 2017). The strains lack *relA* which is primarily responsible for rapid ppGpp synthesis upon nutrient depletion and carry modifications in the bifunctional enzyme SpoT. It was shown that they do not react to the exhaustion of ammonium supply by ppGpp synthesis (Michalowski *et al*., 2017). Strain *E. coli* SR displays no negative phenotypic differences in batch cultivations compared to its parent strain *E. coli* K‐12 MG1655. However, under conditions of ammonium limitation, *E. coli* SR was found to have an elevated specific glucose consumption rate which is beneficial for two‐stage processes involving product formation in the nitrogen limited phase (Jarmander *et al*., 2015; Perez‐Zabaleta *et al*., 2016).

The combination of properties displayed by *E. coli SR* indicates that this strain can potentially be developed as a platform strain for robust scale‐up from lab to production. In this work, we compared the phenotypic and transcriptional responses of *E. coli* SR and its parent strain *E. coli* MG1655 in a two‐compartment scale‐down reactor. We focused our investigation on the regulatory differences between these strains in the response to repeated short‐term stimuli. The primary stirred tank reactor was operated as an ammonium‐limited chemostat while a plug flow reactor simulated a nitrogen starvation zone.

## Results

### Continuous cultivation with periodic nutrient depletion

**Fig. 1:**
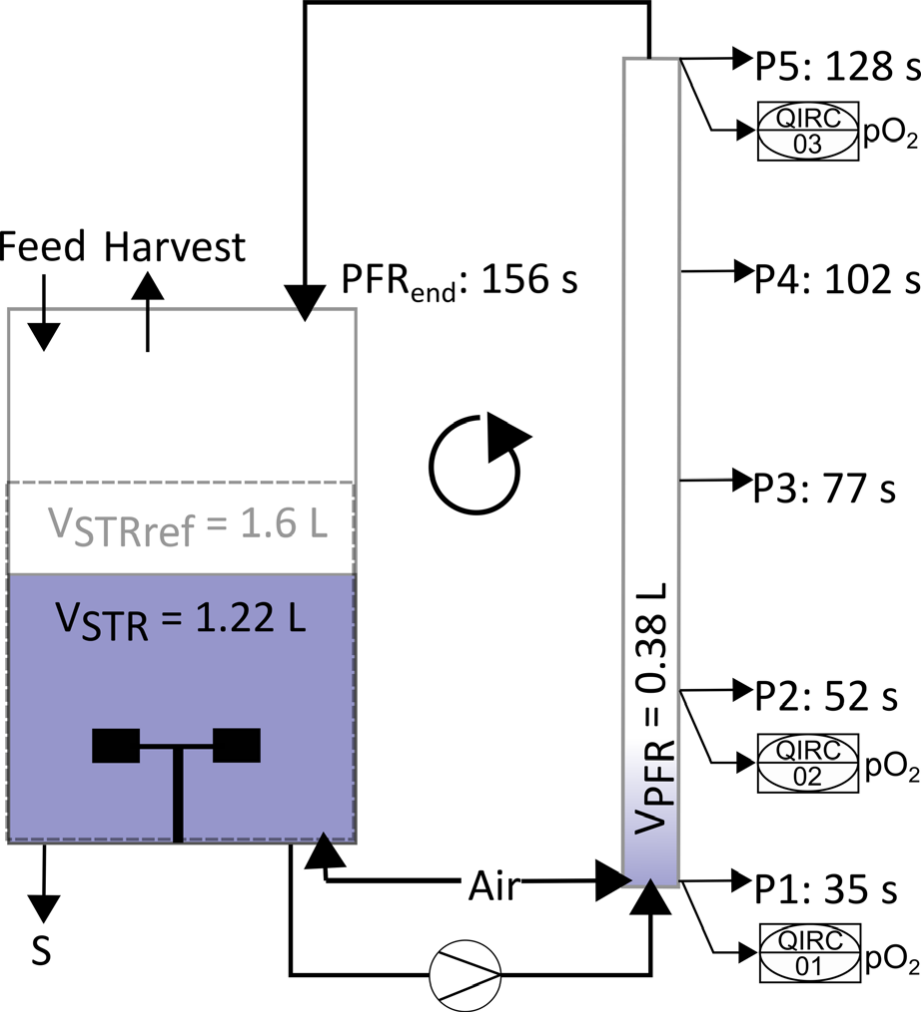
Experimental design of the two‐compartment system. The fermenter consists of a stirred tank reactor (STR) as the primary cultivation vessel and a plug‐flow reactor (PFR) connected by an active pump. The ammonium‐limited chemostat was operated at a dilution rate of D = 0.2 h^‐1^ with the feeding point placed in the STR. The STR served as a limitation zone and the PFR formed a starvation zone. The setup was designed to resolve different timescales of cellular response. Oxygen saturation was measured by three oxygen probes and recorded by the process control system (01, 02, 03). V_STRref_: Reference Volume without connection of PFR (constant volume).

We cultivated *E. coli* SR and *E. coli* MG1655 in two independent continuous fermentations each in a previously described scale‐down reactor consisting of a primary stirred tank reactor (STR) and a secondary plug‐flow reactor (PFR), schematically shown in Fig. 1 (Löffler *et al*., 2016; Simen *et al*., 2017; Ankenbauer *et al*., 2020). *E. coli* SR is a strain with modulated stringent response that was engineered to alleviate the induction of the stringent response and the general stress response upon nutrient depletion (Michalowski *et al*., 2017). The chemostat was operated at a dilution rate of D = 0.2 h^‐1^ and ammonium was chosen as the limiting nutrient. After establishment of a steady state in the STR alone, a reference sample (S0, t = 0 h) was taken and the PFR connected. Periodic passage from the STR (average residence time $\overset{‐}{\tau_{\mathrm{STR}}}=6.2min$) through the PFR (average residence time $\overset{‐}{\tau_{\mathrm{PFR}}}=2.6min$) then created a repeated short nitrogen starvation stimulus. The average residence times represent worst‐case scenarios that are still consistent with mixing studies (Vrábel *et al*., 2000; Noorman, 2011) and the volume ratio STR to PFR was approximately 3:1 to represent existing simulation results (Lapin *et al*., 2006; Haringa *et al*., 2017). The long‐term response of cells was investigated from additional samples taken from the STR shortly after connection of the PFR (S5, t = 5 min) and after establishment of a new steady‐state (S28, t = 28 h) in the two‐compartment cultivation. The short‐term response of cells to the PFR stimulus was monitored by sampling from five ports along the primary axis of the PFR at identical timepoints. Transcript samples for the PFR were taken from port 5 (P5_5 and P5_28).

**Fig. 2:**
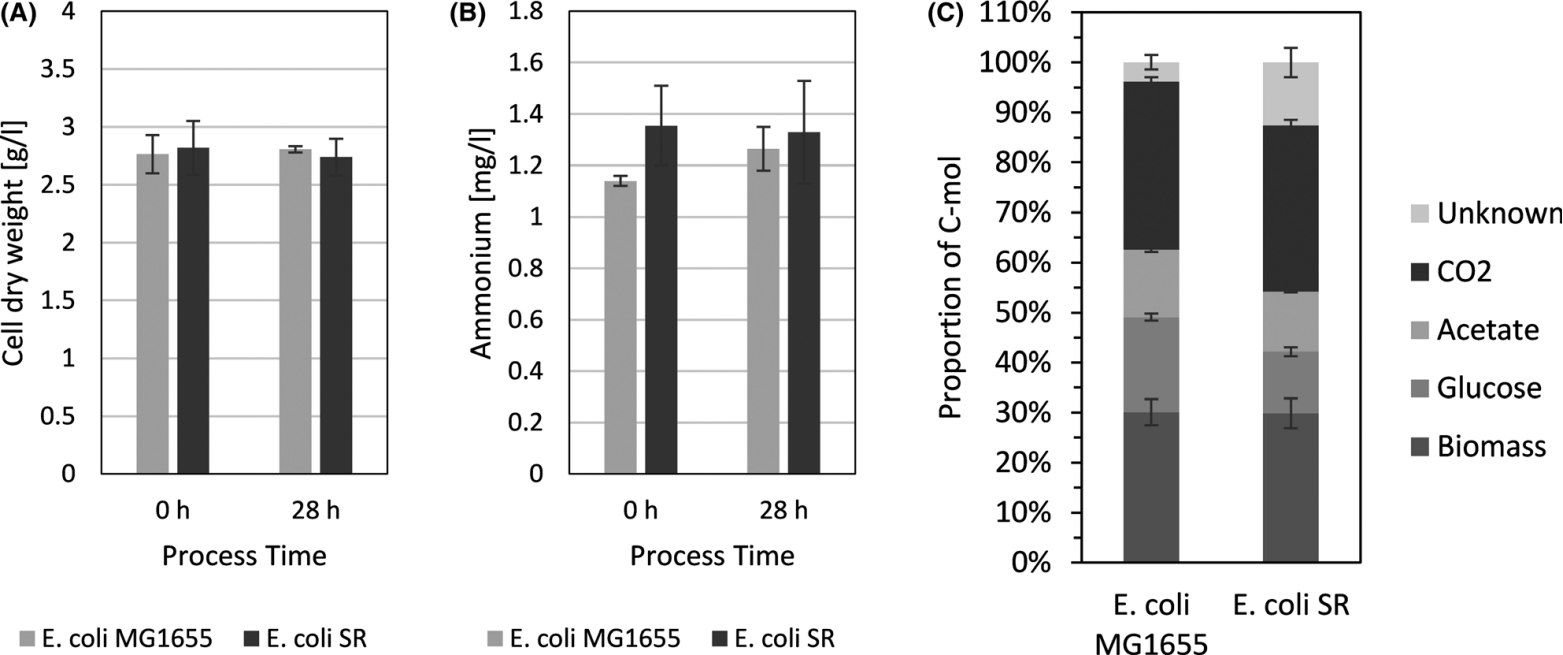
Physiological measurements. A. Cell dry weight. Concentration of cell dry weight after at least 25 h chemostat process before connecting the plug‐flow reactor (0 h) and after 28 h of chemostat process with connected PFR (28 h). B. Ammonium. Concentration of residual ammonium in the supernatant. C. Carbon Balance. Columns show efflux fractions of total C‐mol based on carbon influx. The final fraction represents undetermined dissolved organic substances in the fermentation broth, as measured by the difference of all efflux carbon detected by exhaust gas or total carbon analysis and the sum of the individually measured efflux components. Error bars indicate SEM (*n* = 2) of individual components (A, B and C).

Basic growth and fermentation data confirmed earlier results that there are no detrimental differences in fundamental physiological parameters (Table 1) between *E. coli* MG1655 and *E. coli* SR under nitrogen‐limited conditions (Michalowski *et al*., 2017). There were no statistically significant differences in any parameter (two‐tailed t‐test, p > 0.1). Both strains reached practically identical biomass yields on ammonium and depleted ammonium to equally low levels regardless of process time and PFR action (Fig. 2). The most noteworthy difference between *E. coli* MG1655 and *E. coli* SR was a reduced concentration of excess glucose in the fermentation broth of *E. coli* SR. Consequently, we calculated a lower biomass yield on glucose for *E. coli* SR (Table 1). Under conditions of long‐term nitrogen starvation in batch fermentations *E. coli* SR had previously displayed a relaxation in glucose and nitrogen uptake coupling and we thus suspected an increased specific glucose uptake rate (Michalowski *et al*., 2017). The calculated specific glucose uptake rate was higher for *E. coli* SR, but the difference was not statistically significant in our experiments (two‐tailed *t*‐test, *P*‐value > 0.1). Data from the fermentation broth supernatant showed that both strains converted comparable amounts of substrate into acetate as the primary byproduct. Carbon balancing revealed an increased fraction of unknown substances among the fermentation products of *E. coli* SR which were identified as dissolved organic substances in the fermentation supernatant by total dissolved carbon analysis. The elevated glucose uptake rate of *E. coli* SR likely leads to higher byproduct formation of typical overflow metabolites such as lactate, pyruvate, formate and the regulator 2‐oxoglutarate, all of which are known to accumulate under nitrogen‐limited conditions with glucose excess (Hua *et al*., 2004). Apart from the primary byproduct acetate, individual small carbon byproducts were not measured as the overall total carbon efflux/influx balancing was in good agreement for both strains. Carbon recovery was 101 ± 2 % for *E. coli* MG1655 and 102 ± 1 % for *E. coli* SR indicating that in sum all relevant substances were detected.

**Table 1:** Physiological measurements.

E. coli MG1655	E. coli SR	
$Y_{\mathrm{XN}}\left[\frac{g_{\mathrm{CDW}}}{g_{{\mathrm{NH}}_{4}^{+}}}\right]$	4.63 ± 0.12^a^	4.62 ± 0.27
$Y_{\mathrm{XS}}\left[\frac{g_{\mathrm{CDW}}}{g_{\mathrm{Glucose}}}\right]$	0.32 ± 0.01	0.28 ± 0.01
$c_{\mathrm{Glucose},\mathrm{STR}}\left[\frac{g_{\mathrm{Glucose}}}{l}\right]$	2.07 ± 0.25	1.49 ± 0.06
$c_{\mathrm{Acetate},\mathrm{STR}}\left[\frac{g_{\mathrm{Acetate}}}{l}\right]$	1.39 ± 0.11	1.29 ± 0.14
$q_{{\mathrm{NH}}_{4}^{+}}\left[\frac{g_{{\mathrm{NH}}_{4}^{+}}}{g_{\mathrm{CDW}}*h}\right]$	0.04 ± 0.01	0.05 ± 0.01
$q_{S}\left[\frac{g_{\mathrm{Glucose}}}{g_{\mathrm{CDW}}*h}\right]$	0.63 ± 0.05	0.77 ± 0.14
$q_{\mathrm{Ac}}\left[\frac{g_{\mathrm{Acetate}}}{g_{\mathrm{CDW}}*h}\right]$	0.10 ± 0.01	0.10 ± 0.01
$q_{{\mathrm{CO}}_{2}}\left[\frac{{\mathrm{mmol}}_{{\mathrm{CO}}_{2}}}{g_{\mathrm{CDW}}*h}\right]$	8.73 ± 1.06	9.98 ± 2.23
$q_{O_{2}}\left[\frac{{\mathrm{mmol}}_{O_{2}}}{g_{\mathrm{CDW}}*h}\right]$	9.28 ± 0.47	10.9 ± 2.02
$\mathrm{RQ}\left[\frac{{\mathrm{mol}}_{{\mathrm{CO}}_{2}}}{{\mathrm{mol}}_{O_{2}}}\right]$	0.95 ± 0.16	0.91 ± 0.04
$q_{\mathrm{ATP}}\left[\frac{{\mathrm{mmol}}_{\mathrm{ATP}}}{g_{\mathrm{CDW}}*h}\right]$	29.23 ± 0.62^b^	34.73 ± 6.39
$D\left[\frac{1}{h}\right]$	0.20 ± 0.01	0.21 ± 0.03

^a^ Errors indicate SEM (*n* = 2). All rates were calculated from averaged values collected over the entire STR‐PFR process time.

^b^ Estimated values assuming a P/O‐Ratio of 1.2.

In general, process time and the periodic PFR stimulus hardly affected global process parameters which is in accordance with former observations made in this reactor setup for nitrogen limitation and K‐12 strains (Simen *et al*., 2017). In sharp contrast, we found substantial regulatory differences between the two strains both in the short‐term and in the long‐term transcriptional responses to the periodic starvation stimulus.

### Transcriptomic analysis: Overview

**Fig. 3:**
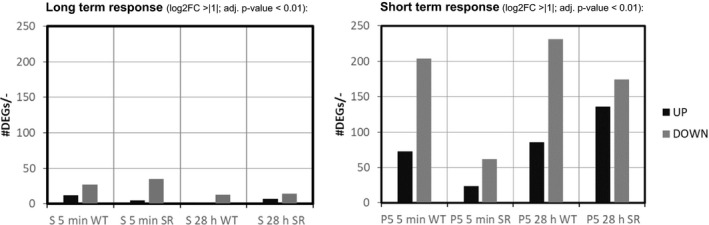
Number of UP (black) and DOWN (gray) regulated genes (DEGs). Long‐term (left) and short‐term (right) response to repeated nitrogen starvation for *E. coli* MG1655 (WT) and *E. coli* SR (SR) and given process times.

RNA‐seq‐based transcriptomic data to examine potentially important genes for the ammonium stress response of *E. coli* WT and *E. coli* SR was analysed. After filtering, 4037 predicted *E. coli* genes remained for further analysis (see Supporting information). The fast tactical transcriptional response to ammonia shortage was determined by comparing PFR port 5 samples to STR samples taken at the same process time points. Long‐term responses were studied by comparing post‐perturbation samples from the STR after 5 min (S5) and 28 h (S28) to the reference sample (S0). The statistical threshold for significance was set for adjusted p‐value < 0.01 and log2FC > |1|. 54 differentially expressed genes (DEGs) (UP: 14, DOWN: 40) formed the long‐term response of *E. coli* MG1655. The short‐term response was more pronounced comprising 837 DEGs (UP: 242, DOWN: 595). *E. coli* SR disclosed a similar number of 61 DEGs for the long term response (UP: 12, DOWN: 49), but substantially less DEGS as short term response (Total: 387, UP: 161, DOWN: 226) (Fig. 3). Log_2_FC values range from −4.69 to 4.96 (WT) and −3.90 to 5.13 (SR). Fig. 3 depicts an overview of transcriptional dynamics outlining the halved response of *E. coli* SR 5 min after repeated nitrogen limited perturbation compared to WT.

**Fig. 4:**
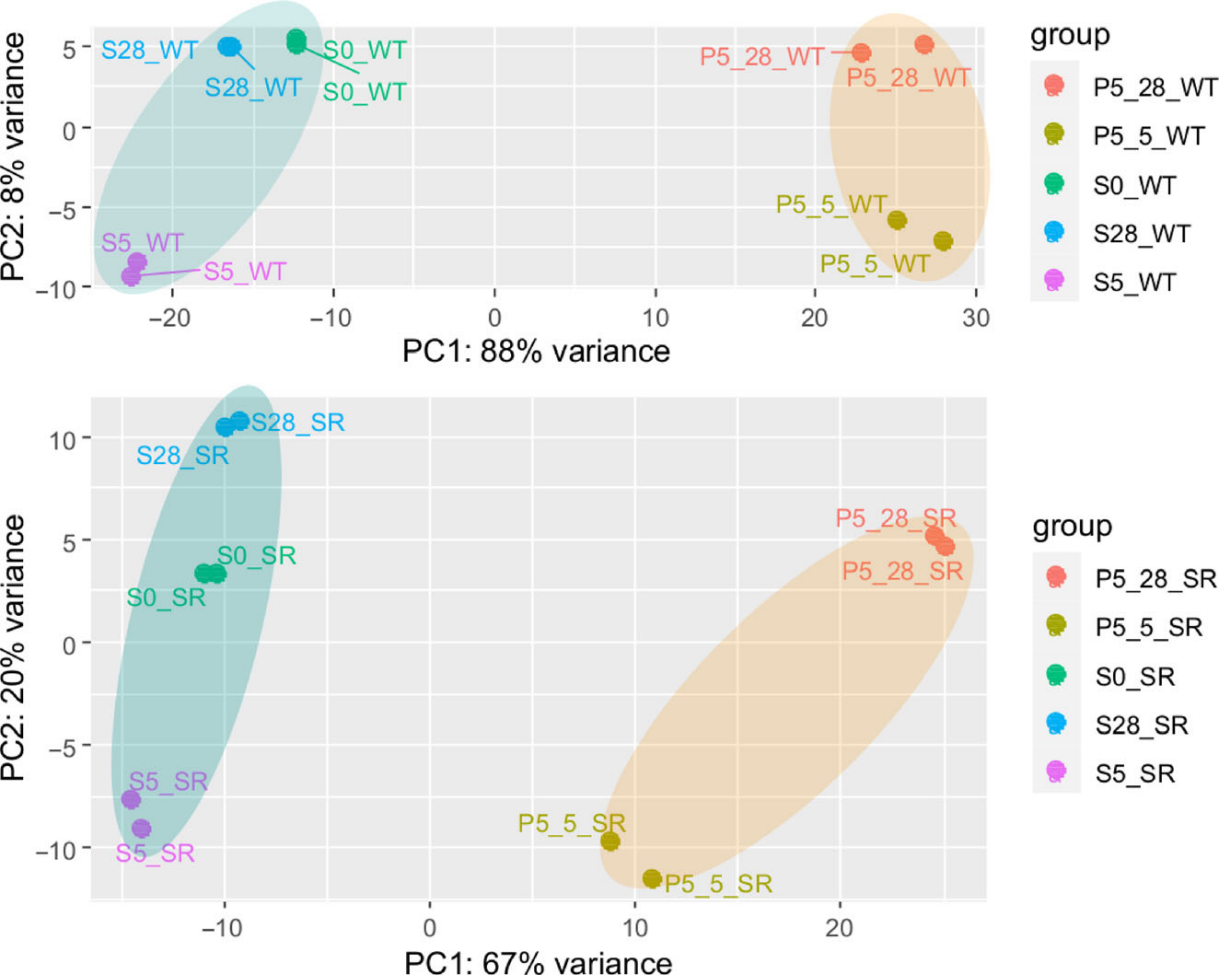
Principal component analysis of transcript data of *E. coli* MG1655 (WT) (top) and *E. coli* SR (bottom) obtained from STR (S) and PFR (port 5, P5) at three process time points (0 h, 5 min, and 28 h). Covered measurement variance of each principal component (PC) is indicated. Ellipses cluster samples of STR and PFR. PC1 accounts for ‘sample port location’, PC2 for ‘process time’.

Figure 4 shows that the multi‐transcript response of each strain could be well described by 2‐dimensional PCA covering 96% and 87% of total variance for *E. coli* WT and *E. coli* SR, respectively. Notably, biological duplicates were found in close proximity. PC1 accounts for the sample port location, PC2 for the time course. Unique and clearly distinguishable differences between STR and PFR transcript patterns were observed already after 5 min of repeated nitrogen starvation for both strains (Figure 4, A1). In particular, principal component 1 (PC1) disclosed major differences between the samples of each strain accounting for 88% and 67% regarding *E. coli* WT and *E. coli* SR, respectively. The PCA finding is in agreement with the reduced number of DEGs observed for *E. coli* SR. The impact of PC2 is more pronounced for *E. coli* SR although almost identical numbers of DEGs were found as long‐term response in both strains. However, given the low impact of PC1 for *E. coli* SR, similar DEG values affect the relative principal component analysis stronger.

As long‐term responses of both strains were similar (see Appendix: Supporting information) and weaker than short‐term responses (Fig. 4) further analysis focused on short‐term transcript patterns. Notably, changes between long‐ and short‐term responses of both strains were dominated by counteracting transcript dynamics resetting perturbations after PFR passages (MG1655: 5 min and 28 h). Observations are in line with similar findings (Chang *et al*., 2002). Additional differences were found in the upregulation of carbohydrate transport (SR: 5 min) and catabolic processes (SR: 28 h) (see Fig. A5 and A6).

### Regulatory response to short‐term ammonium limitation

**Fig. 5:**
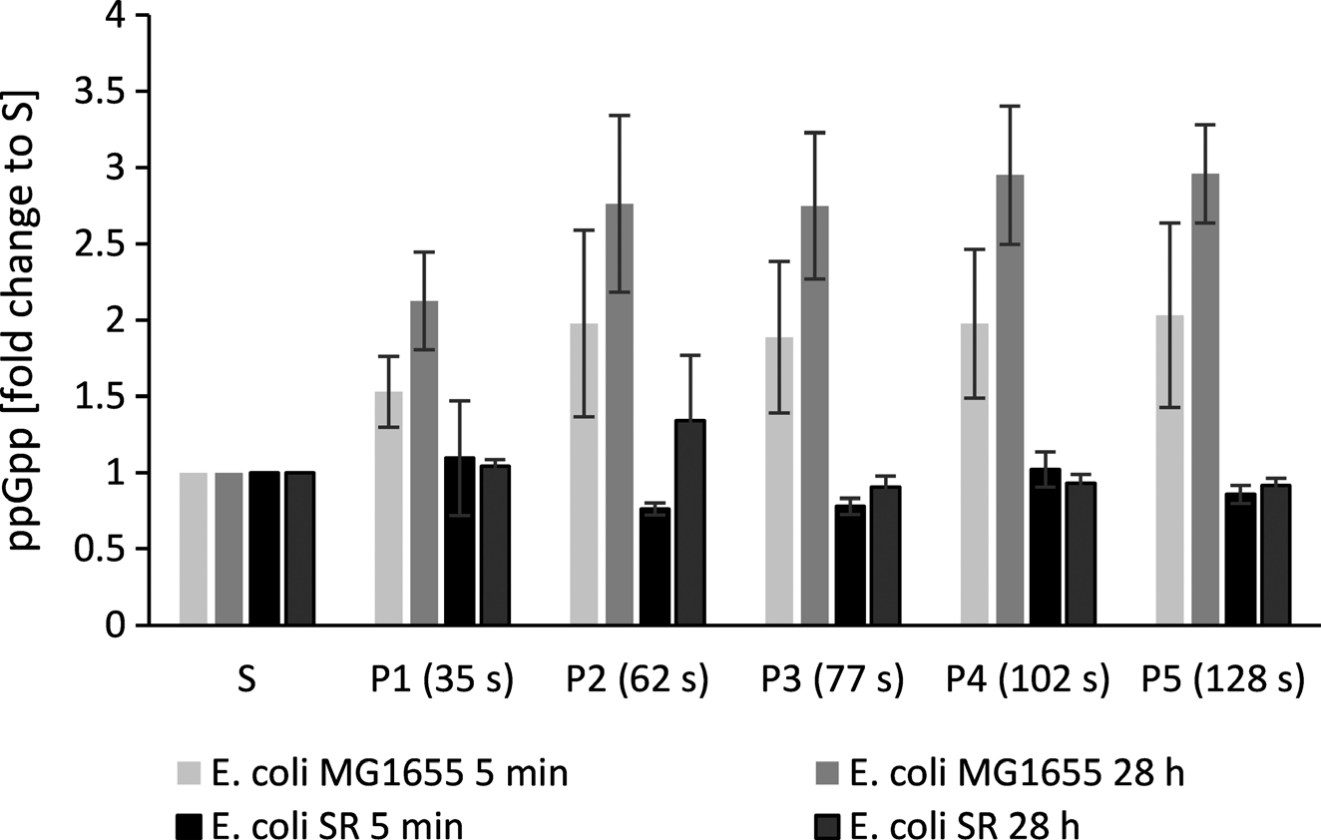
Alarmone accumulation along the PFR. Concentration of ppGpp measured from samples drawn along the plug flow reactor (P1 to P5) relative to the concentration measured in the stirred tank reactor (S, all values set to 1) for *E. coli* MG1655 (WT) and *E. coli* SR (SR). Error Bars represent SEM (*n* = 2).

Preceding investigations of *E. coli* K‐12 strains in STR‐PFR scale‐down reactors revealed the rapid accumulation of the alarmone ppGpp upon entry into the nutrient limited zone under both glucose and ammonium limitation (Löffler *et al*., 2016; Simen *et al*., 2017). Concomitantly, an extensive transcriptional reprogramming of cells occurred. In standard batch fermentations *E. coli* SR in turn did not react to ammonium depletion by ppGpp synthesis (Michalowski *et al*., 2017). We therefore measured intracellular ppGpp levels from samples taken from the five ports of the PFR along its primary axis (Fig. 5). During the PFR passage *E. coli* MG1655 accumulated ppGpp to levels 2 – 3 fold higher than measured in the STR, displaying the same behaviour as previously observed for the closely related K‐12 strain *E. coli* W3110 (Simen *et al*., 2017). In contrast, *E. coli* SR had no elevated levels of ppGpp at any point during the PFR passage regardless of process time. These results complement previous findings for the case of repeated short stimuli and confirm the strain’s resilience to ammonium exhaustion.

**Fig. 6:**
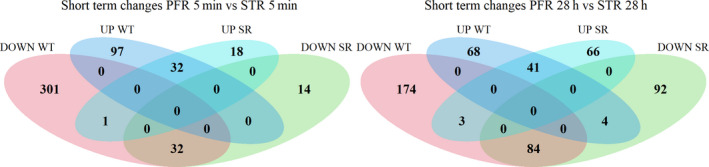
Venn diagrams representing partially overlapping sets of DEGs of *E. coli* MG1655 (WT) and *E. coli* SR. The number of significantly up‐ (UP) and downregulated (DOWN) genes in each set is indicated by numbers. Left: Short‐term responses 5 min after PFR connection. Right: Short‐term responses 28 h after PFR connection. Complete gene lists of the Venn diagrams are available in the supplementary data.

**Fig. 7:**
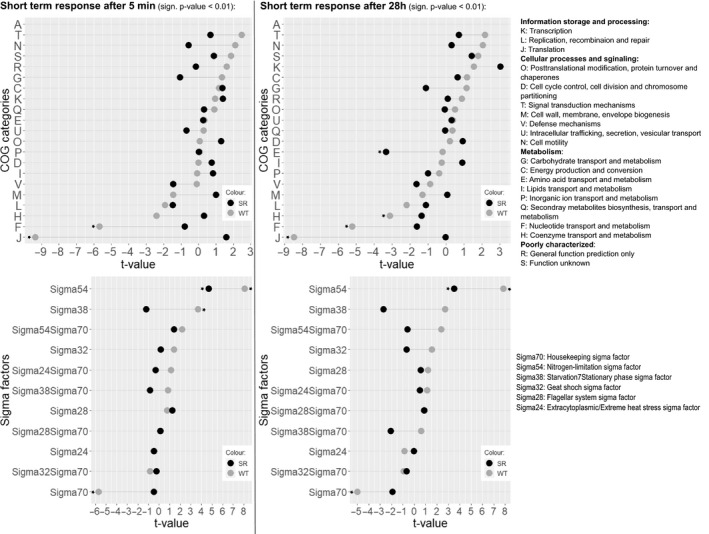
Top: Transcriptional patterns grouped into COG categories of *E. coli* MG1655 (WT) and *E. coli* SR (SR). Left: short‐term patterns to the PFR stimulus 5 min after PFR connection. Right: short‐term patterns to the PFR stimulus 28 h after PFR connection. Bottom: Sigma factor activities of *E. coli* MG1655 (WT, grey) and *E. coli* SR (SR, black). Left: Short‐term response to the PFR stimulus 5 min after PFR connection. Right: Short‐term response to the PFR stimulus 28 h after PFR connection. Significant categories are indicated with an asterix.

Based on these encouraging findings, we focused our investigation on the short‐term transcriptional response of both strains along the PFR axis. We compared data from samples drawn from port 5 of the PFR to samples drawn from the STR at identical process time points. Short‐term changes revealed a significantly different response of *E. coli* SR compared to *E. coli* MG1655 not only in the amount of DEGs (Fig. 3), but also in the function of these genes (Fig. 4, 6). To elucidate patterns in the transcriptional responses, we searched for common DEGs, investigated the behaviour of gene clusters of orthologous groups (COGs), and compared sigma factor (σ) activities. The gene expression patterns of each strain individually were assigned to 21 functional categories based on the COG database (Tatusov *et al*., 2003). In total 3532 of the 4037 genes (87.5%) could be annotated to COG. For each COG category, the resulting t‐values are represented in a lollipop plot (Fig. 7). Significant changes were defined with a FDR‐corrected p‐value < 0.01. Furthermore, the activation and deactivation of sigma factors over time were investigated (Fig. 7). In this case, 3935 out of 4037 genes could be assigned to the sigma factor‐gene interaction database from RegulonDB (Santos‐Zavaleta *et al*., 2019).

After the first 5 min of PFR action *E. coli* MG1655 and *E. coli* SR exhibited substantially different transcriptional responses. The strains had only 64 DEGs in common, split equally between up‐ and down regulation (Fig. 6 left). Hence, these genes mirror the transcriptional response to short‐term starvation irrespective of a functional stringent response, in which 14 out of the 32 common upregulated genes are associated with the Ntr‐reponse (e.g. *glnK*, *amtB*, *glnAHPQ*, *rutA*). Downregulated genes consist of genes responsible for amino acid biosynthesis (e.g. *argCF*, *metABFINR*) and other cellular functions such as DNA cleavage, transporters and oxidoreductases. The only oppositely regulated gene was *guaC* coding for the GMP reductase GuaC. Transcriptional control of the *guaC* promoter by the stringent response was proposed after its initial discovery and is clearly supported by our data (Andrews and Guest, 1988). Individual, strain‐specific short‐term regulation was observed for 398 (*E. coli* MG1655) and 32 (*E. coli* SR) specific DEGs after 5 min, clearly demonstrating the effect of the stringent response on the *E. coli* transcriptome.

Gene expression along the PFR after 28 h of PFR action differs strongly from the early response. 125 DEGs, mostly downregulated, are shared by both strains and the number of individually regulated genes is similar with 242 genes for *E. coli* MG1655 and 158 genes for *E. coli* SR (Fig. 6 right). Additionally, seven genes are oppositely regulated. Three of them (*tolQ, guaC, purM*) are upregulated in *E. coli* SR and downregulated in *E. coli* MG1655. These genes correspond to cell envelope integrity during cell division (Gerding *et al*., 2007), nucleotide metabolism (Kanjee *et al*., 2012) and purine *de novo* biosynthesis (Mueller *et al*., 1999). While purine *de novo* biosynthesis is actively inhibited by ppGpp via inhibition of GuaB, GTP synthesis solely originates from purine salvage pathways with *xdhA* significantly increased in *E. coli* MG1655 (Xi *et al*., 2000). The residual four oppositely regulated DEGs (*csiD, glnL, lhgO, yeaH*) predominantly play a role in the adaptation to nitrogen starvation and except for *glnL* are known to be induced by ppGpp. NtrB encoded by *glnL* is an essential part of the Ntr response cascade to nitrogen starvation and *yeaG* positively impacts *rpoS* transcription and translation under prolonged nitrogen starvation (Brown *et al*., 2014). Despite these differences in adaption to nitrogen limitation, we observed no alterations in the uptake or utilization of ammonium which indicates that the additional regulatory adaptions of *E. coli* MG1655 are irrelevant in the context of a bioprocess.

Transcriptional patterns could be identified by functional enrichments of groups based on COG categories and sigma factor activities. COG groups J (Translation, ribosomal structure, and biogenesis) and F (nucleotide transport and metabolism) were significantly down regulated as part of the stringent response of *E. coli* MG1655 after both 5 min and 28 h (Fig. 7). For the 28 h sampling point group H (coenzyme transport and metabolism) was also significantly downregulated. As already indicated by the oppositely regulated genes (Fig. 7), σ54‐mediated genes responsible for the activation of the Ntr stress response including *yeaG/H* via NtrBC were induced in *E. coli* MG1655, as well as the σ38 regulon as part of the general stress response (Brown *et al*., 2014; Figueira *et al*., 2015) (Fig. 7). Due to the limited amount of RNA‐Polymerase (RNAP) core enzymes, σ70 competes with σ54, resulting in an antiproportional expression of their mediated genes (Jishage *et al*., 1996). In contrast, *E*. *coli* SR only increased the expression of genes regulated by σ54 after 5 min and no significant COG category was identified at this time‐point. The absence of the stringent response in *E. coli* SR is clearly visible in an overall dampened regulatory response. The only significantly regulated group is E (amino acid transport and metabolism) after 28 h of PFR action, and the significantly downregulated genes in this group are predominantly ABC‐transporters.

**Fig. 8:**
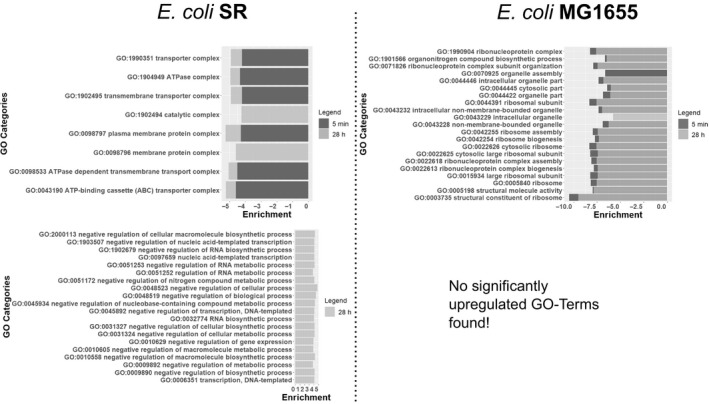
Significant GO categories after 5 min and 28 h of both *E. coli* SR (left) and *E. coli* MG1655 (right). Downregulated categories are arranged at the top and upregulated GO terms at the bottom. 5 min: Short‐term response of *E. coli* SR (left) and *E. coli* MG1655 (right) after 5 min of PFR action. Only the Top 20 out of 102 significantly downregulated categories are shown. Neither strain had significantly upregulated categories for this time‐point. 28 h: Short‐term response of *E. coli* SR (left, light grey) and *E. coli* MG1655 (right, light grey) after 28 h of PFR action. For *E. coli* SR only the Top 20 out of 24 significantly upregulated categories are shown. For *E. coli* MG1655 only the Top 20 out of 95 significantly downregulated categories are shown. No significantly upregulated categories were found for this time‐point.

To unravel more detailed patterns in the transcriptional responses we assigned genes to the up‐to‐date gene ontology (GO) gene sets using GAGE (Luo *et al*., 2009). 3345 out of 4037 genes (83%) could be mapped to GO Terms. As shown in Fig. 3 the majority of significant DEGs for *E. coli* MG1655 were downregulated. This is mirrored by the results of the identified top 20 GO categories which were uniformly down‐regulated (Fig. 8). *E. coli* MG1655 predominantly downregulated genes related to ribosomal biosynthesis and translation after 5 min and 28 h as expected for a stringent phenotype (Fig. 8). These transcriptional changes are counteracted in the long‐term response observed from the STR (Fig. A3 to A6) which indicates looping induction and repression of the genes. Patterns from *E. coli* SR were less pronounced and grouped differently. After 5 min we observed decreasing gene expression of ATP‐demanding processes such as ABC transporters and ATPase complexes (Fig 8). After 28 h the PFR passage mainly induced an increased negative regulation of transcription and metabolic processes (Fig. 8). Care must be taken in the interpretation of this group though. General categories affecting transcription (GO:0006351, GO:0045892, GO:0097659, GO:1903507) or RNA processes (GO:0032774, GO:1902679, GO:0051253, GO:0051252) are represented as simultaneously negatively and positively regulated. Moreover, all negative regulators included in these terms, such as members of the CRP family, are also capable of positive regulation. Other negative regulation categories involve genes which actively inhibit translation and belong to SOS signals like DNA damage, prevention of cell division and programmed cell death (PCD). *E. coli* SR thereby focuses on σ38 regulated genes, as well as toxin and antitoxin systems (*mazEF* and *mqsRA*) possibly resulting in arrested growth and a dormant cell state or even PCD. As growth arrest is usually a primary outcome of the stringent response, which is absent in *E. coli* SR, we hypothesize that this pattern might provide an alternative way for *E. coli* SR to achieve cell cycle arrest.

In summary, the short‐term response transcriptional patterns of *E. coli* MG1655 were extensive and dominated by the stringent response and the Ntr regulon. The major activated sigma factors were σ54 and σ38. Overall, the transcription of ribosomal genes and other genes necessary for growth was inhibited, while genes involved in the transport and fixation of ammonia were induced. Our observations reflect well‐known regulatory patterns exerted by *E. coli* K‐12 when facing nitrogen starvation (Chang *et al*., 2002; Traxler *et al*., 2008; Traxler *et al*., 2011; Simen *et al*., 2017; Wang and Levin, 2009). In contrast, the transcriptional short‐term response of *E. coli* SR is dampened both in the number of DEGs and the patterns observed, especially shortly after connection of the PFR. The only significantly activated sigma factor is σ54 indicating a functional but attenuated Ntr response in the absence of ppGpp accumulation. Adaptation to ongoing starvation was possibly attempted via negative regulation of metabolic processes and SOS pathways.

## Discussion

In the present study, we investigated the regulatory responses of the stringent response mutant strain *E. coli* SR when exposed to repeated short starvation stimuli in a scale‐down reactor. The comparison with its wild‐type parent *E. coli* MG1655 unravelled dampened regulatory patterns which are potentially beneficial for the application of *E. coli* SR in industrial large‐scale reactors. The reduced regulatory patterns might be beneficial for heterologous protein expression as well as the production of small molecules as less interference with engineered metabolic pathways may occur and energy otherwise spent for adaptive responses is available for product formation.

An important finding of our study is that despite the regulatory differences *E. coli* SR displayed no dysfunctionalities in handling the shortage of ammonium. *E. coli* SR reached the same biomass yield on ammonium as *E. coli* MG1655 both with and without PFR action. Moreover, both strains depleted ammonium to comparable levels of about 1.2 mg l^‐1^ or 67 µmol l^‐1^, well in line with previously reported values for *E. coli* K‐12 strains in nitrogen limited chemostats (Hua *et al*., 2004). The low remaining ammonium concentration indicates that uptake in both strains is mediated actively by AmtB with K_m_ = 0.8 mM (Williamson et al., 2020) and incorporation is accomplished by the GS‐GOGAT System with GS K_m_ = 0.1 mM (Alibhai and Villafranca, 1994). This is supported by our transcriptional data which revealed that *amtB*, *gltB* and *gltD* were significantly enriched for both strains over all time‐points. Transcripts of *glnA* were also always significantly enriched except for the time point 28 h of *E. coli* SR. Concomitantly, we identified transcriptional patterns typical for the σ54‐ and NtrBC‐mediated responses to nitrogen starvation (Reitzer, 2003). 13 out of 21 known NtrC‐regulated operons (Brown *et al*., 2014) were induced at PFR port 5 in *E. coli* MG1655 at all time points (Table S2). For *E. coli* SR, the Ntr response was slightly reduced, with 9 out of 21 operons induced (Table S3) and lower overexpression of σ54 transcribed genes. These findings lead to the conclusion of an active, but diminished Ntr response of *E. coli* SR that still allowed fully functional ammonium assimilation. Additionally, the energy consumption as maintenance add‐on for both strains was calculated according to Löffler *et al*. (2016) assuming *de novo* synthesis of all upregulated DEGs over the whole process time (28 h). The resulting energy savings of *E. coli* SR due to weaker transcriptional response added up to around 46.5 %. In terms of microbial productivity, the reduced maintenance demand potentially increases the amount of available ATP for biomass‐specific productivities and improves cell fitness.

In a previous study, a significantly elevated specific glucose consumption rate under ammonium limitation was observed in *E. coli* SR (Michalowski *et al*., 2017). Similarly, we observed reduced excess glucose and the accompanying formation of dissolved byproducts in the fermentation supernatant. In *E. coli* K‐12 strains, the consumption of glucose is usually tightly coupled to the availability of nitrogen on the level of metabolite control by the interaction of 2‐oxoglutarate with PtsI (Doucette *et al*., 2011). The exact mechanism by which coupling of nitrogen and glucose uptake rates are relaxed in *E. coli* SR is not clear as the strain is isogenic to *E. coli* MG1655 except for the deletion of *relA* and the modifications in *spoT*. However, we found an increased transcription of *ptsI*, *ptsH* and *ptsG* in *E. coli* SR compared to *E. coli* MG1655 (Table S6). Artificially increased expression of *ptsI* has been shown to increase specific glucose uptake rates in nitrogen limited conditions (Chubukov *et al*., 2017). We presume that the increased glucose uptake rate in *E. coli* SR might be caused by deregulated expression of *ptsI*, potentially connected to the absence of the stringent response by the action of CRP whose transcription is negatively regulated by ppGpp (Johansson *et al*., 2000). It remains to be clarified whether *E. coli* SR has altered cytoplasmic 2‐oxoglutarate levels or the action of ppGpp influences the coupling of glucose consumption to nitrogen availability, potentially by the proposed mechanism. Increased specific glucose uptake rates in conjunction with higher respiratory activity have also been observed in *E. coli* MG1655 subjected to repeated glucose feast‐famine cycles (Vasilakou *et al*., 2020). Future studies should thus examine how *E. coli* SR reacts to varying availability of glucose or other carbon sources.

In view of these differences in carbon metabolism, we hypothesized that biological energy availability might be unequal for *E. coli* MG1655 and *E. coli* SR. From oxygen and glucose uptake rates the specific ATP production rate q_ATP_ was estimated (Table 1). q_ATP_ greatly depends on the effective P/O ratio and current scientific consensus estimates realistic P/O ratios between 1.0 and 1.5 for *E. coli* (Noguchi *et al*., 2004; Szenk *et al*., 2017). For our estimations of q_ATP_ we assumed a conservative P/O ratio of 1.2 and 2 moles of ATP per mol glucose from glycolysis. The result indicates that *E. coli* SR might have an increased availability of ATP compared to its wild‐type parent under the applied experimental conditions. Given that the respiratory capability and thus the ATP production capability of K‐12 strains is not exhausted at a dilution rate of D = 0.2 h^‐1^ it appears that the increased glycolytic flux to byproducts displayed by *E. coli* SR was also not a result of increased energy demand. Moreover, increased glucose uptake has been reported previously for *E. coli* SR under conditions of ammonia limitation despite high adenylate energy charge (Michalowski *et al*., 2017). Carbon and redox homeostasis at elevated glycolytic flux would then be maintained by byproduct excretion and increased respiration, possibly involving the dissipation of surplus energy by uncoupling of the electron transport chain (Bekker *et al*., 2009).

Nitrogen limitation inducing the stringent response is a well‐documented phenomenon in *E. coli*. Multiple previous studies predominantly observed heavily increased gene expression corresponding to amino acid transport and metabolism (Barker *et al*., 2001; Durfee *et al*., 2008; Traxler *et al*., 2008; Traxler *et al*., 2011; Brown *et al*., 2014; Simen *et al*., 2017). Conversely, we observed almost equally distributed up‐ and downregulated genes for amino acid transport and metabolism (see Supporting information: Transcriptomics), which was only reported by few research groups (Chang *et al*., 2002; Traxler *et al*., 2008). As a result, no overall significant statistical trend was detectable for this category (Fig. 7). We suggest that the individual operons do not solely respond to ppGpp, but rather depend on other signals and regulatory networks which were not found to be significantly expressed in this study such as the Lrp regulon. Additionally, caution is advised when comparing transcriptomic analyses originating from different studies as they greatly depend on the transcriptional reference state and thus the details of the experimental design.

In general, the amount of DEGs of *E. coli* K‐12 MG1655 was similar to the numbers found in the analogous study of Simen *et al*. (2017) who employed the closely related *E. coli* K‐12 W3110 confirming the validity of our data. The amount of DEGs is also less than observed during the related study of glucose starvation by Löffler *et al*. (2016) which points towards significant potential of *E. coli* SR to preserve energy in glucose starvation conditions. An interesting difference to the former studies in this scale‐down reactor setup is the absence of increased motility in the STR after PFR connection (Löffler *et al*., 2016; Simen *et al*., 2017). Our dataset contains no upregulated flagellar or sigma factor 28 mediated gene patterns from the STR at any time‐point (Fig. 7). We first hypothesized that the cause might be genetic differences affecting motility which are well documented between MG1655 and W3110 and even between different MG1655 isolates (Barker *et al*., 2004; Hayashi *et al*., 2006). However, sequencing of our MG1655 isolate revealed the presence of the canonical IS‐1 insertion upstream of *flhD* which confers motility and our MG1655 isolate displayed vivid spreading in motility agar (Supporting information, Fig. A7). An alternative explanation could be derived by the interplay of quorum sensing and flagellar regulation through the action of autoinducer‐2 (AI‐2) and the motility quorum sensing regulator MqsR. While transcript levels of *luxS* (LuxS synthesizes Al‐2) remain unchanged, the expression of *mqsR* is significantly enriched at PFR port 5 and MqsR is known to induce the flagellar synthesis cascade (González Barrios *et al*., 2006). However, cell dry weight (CDW) was always below 3 g l^‐1^ in our experiments whereas Simen *et al*. worked with around 10 g l^‐1^ CDW. Higher biomass should lead to increased AI‐2 levels and may cause a preconditioned phenotype that rapidly initiates flagellar biosynthesis when encountering nutrient stress. Thus, rapid induction of motility genes might become more pronounced during high cell density processes in large‐scale reactors and remains to be examined in further studies. Additionally, as introduced by Löffler *et al*.(2016) during glucose fluctuation, genes of the category cell motility were identified as one of the most prominent energy consumers and might therefore be candidates for genome reduction (Löffler *et al*., 2016).

Analysis of gene expression patterns (Fig. 7 and 8) revealed that both strains individually adapted to repeated nitrogen starvation. *E. coli* MG1655 adjusted by utilizing the ppGpp‐mediated general stress response including activation of toxin/antitoxin (TA) systems like *mqsRA* and *mazEF*. This strategy intends to arrest the cell cycle and form persister cells (Balaban *et al*., 2004). Persister cell formation is not yet fully understood and usually only involves a small fraction of cells (Chowdhury *et al*., 2016; Gerdes and Maisonneuve, 2012; Korch *et al*., 2015). Thus, it seems to be only of minor importance for industrial processes but some persister genes affect persister level due to altered growth rates rather than contributing to a mechanism of cell cycle arrest and might have a significant impact on bioprocess performance (Allison *et al*., 2011). Nonetheless two common dependencies affecting persister formation, ppGpp and TA systems, are known which is in line with our findings (Aizenman *et al*., 1996; González Barrios *et al*., 2006; Chowdhury *et al*., 2016; Sun *et al*., 2017; Wang and Levin, 2009). Persister formation benefits from increased ppGpp concentrations but is still possible at lower rates in the absence of ppGpp by proteins which simply reduce growth (Chowdhury *et al*., 2016). The nucleotide pyrophosphohydrolase MazG which is negatively regulated by the *mazEF* system is able to initiate cell cycle arrest and was significantly upregulated in *E. coli* SR after 28 h (Lee *et al*., 2008). Additionally, *E. coli* SR initiated negative regulation of transcription, translation and cell division processes as part of the SOS response (Fig. 8). Most likely, the SOS pathways were activated due to ongoing DNA replication during starvation conditions which might ultimately result in DNA damage and inhibited cell division (Bi and Lutkenhaus, 1993; Joseleau‐Petit *et al*., 1999; Traxler *et al*., 2008). As part of the SOS response and as a key gene involved in filamentation *sulA* was significantly upregulated in *E. coli* SR. SulA inhibits the initiation of cellular division by repressing the assembly of FtsZ into the Z ring (Huisman *et al*., 1984; Fonville *et al*., 2010). Simultaneously with the overexpression of *sulA*, *lexA* was significantly increased which acts as a major repressor of SOS signals. LexA regulates the response strength and is actively involved in the occurrence of persister cells in bacterial populations (Butala *et al*., 2011). These results indicate a coordinated and rather complex SOS response in *E. coli* SR to form persister cells which is not yet fully understood.

The natural regulation of *E. coli* has evolved towards optimality in its lifestyle as a gut bacterium and is not honed for the demands of a large‐scale bioprocess. The absence of the stringent response and the conservation of the ability to grow efficiently in minimal medium suggest that *E. coli* SR has the potential to become a platform strain for applications in large‐scale reactors. Our transcriptional analysis shows that the short‐term response of *E. coli* SR to ammonium depletion is dampened but a functional Ntr/σ54 response remains. Regarding glucose‐limited fermentations, we hypothesize that *E. coli* SR has significant potential to preserve energy in such conditions since the regulatory responses are usually even more pronounced and centred around the stringent response (Hardiman *et al*., 2007; Löffler *et al*., 2016). We therefore propose to confirm the suitability of *E. coli* SR for large‐scale applications in multi‐compartment scale‐down reactors employing exemplary small‐molecule production scenarios. These should include standard glucose‐limited fed‐batches as well as ammonium limited fed‐batches with a prolonged nitrogen‐limited production phase to exploit its elevated glucose consumption.

## Experimental procedures

### Bacterial strains and media

Strains *E. coli* MG1655 or *E. coli* SR were used in all experiments (Table 2).

**Table 2:** Bacterial Strains used in this study.

Strain	Genotype/strain information	Reference
*Escherichia coli* K‐12 MG1655 (“wild type” strain, abbrev. WT)	F^−^, λ^−^, *ilvG* ^−^, *rfb*‐50, *rph*‐1	Michalowski et al. (2017)
*Escherichia coli* SR	MG1655 ∆*relA*, *spoT*[R290E;K292D]	Michalowski et al. (2017)

2xYT agar plates were prepared by autoclaving 16 g l^‐1^ tryptone, 10 g l^‐1^ yeast extract, 5 g l^‐1^ NaCl and 18 g l^‐1^ agar‐agar dissolved in demineralized water. Minimal medium for precultures consisted of 4 g l^‐1^ glucose, 0.96 g l^‐1^ NaH_2_PO_4_⋅2H_2_O, 3.51 g l^‐1^ K_2_HPO_4_, 2.4 g l^‐1^ (NH_4_)_2_SO_4_, 0.01 g l^‐1^ thiamine hydrochloride and 0.2% (V/V) trace elements stock solution. Minimal medium for batch cultivation in the bioreactor consisted of 19 g l^‐1^ glucose, 1.50 g l^‐1^ NaH_2_PO_4_⋅2H_2_O, 3.9 g l^‐1^ K_2_HPO_4_, 5.7 g l^‐1^ (NH_4_)_2_SO_4_ and 0.2% (V/V) trace elements stock solution. 200 µl of antifoaming agent Struktol J647 (Schill + Seilacher, Hamburg, Germany) was added to the batch medium prior to inoculation. Minimal medium for continuous chemostat cultivation in the bioreactor consisted of 11.4 g l^‐1^ glucose, 1 g l^‐1^ NaH_2_PO_4_⋅2H_2_O, 2.6 g l^‐1^ K_2_HPO_4_, 2.28 g l^‐1^ (NH_4_)_2_SO_4_ and 0.2% (V/V) trace elements stock solution. Throughout the chemostat phase 50 µl/h of antifoaming agent Struktol J647 were added continuously to the fermentation medium. The composition of trace element stock solution was 4.175 FeCl_3_⋅6H_2_O, 0.045 g l^‐1^ ZnSO_4_⋅7H_2_O, 0.025 g l^‐1^ MnSO_4_⋅H_2_O, 0.4 g l^‐1^ CuSO_4_⋅5H_2_O, 0.045 CoCl_2_⋅6H_2_O, 2.2 g l^‐1^ CaCl_2_⋅2H_2_O, 50 g l^‐1^ MgSO_4_⋅7H_2_O and 55 g l^‐1^ sodium citrate dihydrate. Stock solutions of salts, trace elements and glucose were autoclaved separately, and stock solutions of thiamine hydrochloride were filter sterilized and stored at 4°C. All compounds were combined just before the experiments to prevent possible aging of media.

### Bioreactor setup

Cultivations were carried out in a two‐compartment scale‐down reactor. The primary reactor was a stirred tank reactor (STR), and a plug flow reactor (PFR) was used as the secondary compartment mimicking a starvation zone. The plug flow reactor was connected to the stirred tank reactor after establishment and sampling of a steady state in the chemostat phase. The basic technical setup has been characterized previously (Löffler *et al*., 2016; Simen *et al*., 2017). Minor modifications to the original setup have been made and are described elsewhere (Ankenbauer *et al*., 2020).

The primary reactor was a 3 l bioreactor (Bioengineering, Wald, Switzerland) equipped with flow baffles and two six‐blade Rushton type impellers operated at 1000 rpm. A constant aeration rate of 2.0 standard litres of ambient pressurized air per minute was employed and the system operated at a total pressure of 1.5 bar. Temperature was monitored by a platinum resistance thermometer and regulated by electrical heating or water cooling. Temperature was set to 28–30°C for the batch phase and to 37°C for the continuous chemostat phase. The reactor was equipped with a pH sensor (Mettler Toledo, Columbus, USA) to control pH and a pO2 sensor for monitoring dissolved oxygen tension (PreSens, Regensburg, Germany). During all fermentation stages pH was set to 7.0 and regulated by automated addition of 3 M NaOH or 2.5 M H_3_PO_4_. Dissolved oxygen tension was not regulated but maintained values above 70% saturation to 1.5 bar ambient air throughout the entire cultivation. In the exhaust gas stream, the concentration of oxygen and carbon dioxide was measured by gas sensors (BlueSens, Herten, Germany). During the chemostat phase the feed was constantly added to the reactor by a peristaltic pump (Watson‐Marlow, Falmouth, UK). The feed flow was monitored by a balance recording the weight of the stirred feed barrel and manually adjusted if necessary. The harvesting pump operated as a slave pump set to maintain a constant weight of the bioreactor. For this purpose, the stirred tank reactor was installed on a balance as well.

The secondary compartment was a plug‐flow reactor with an inner tube diameter of 20 mm and a total volume of approximately 380 ml. Five ports along the primary axis were used to take samples throughout the cultivation. Oxygen saturation in the PFR was monitored close to ports P1, P2 and P5 and additional aeration of 0.15 standard litres per minute was provided next to port P1 to ensure levels above 30% saturation to ambient air conditions throughout the entire PFR passage. Temperature in the PFR was maintained at 36–37°C by water heating and isolation material. A diaphragm metering pump (Sigma/1, ProMinent, Heidelberg, Germany) was used to transfer biosuspension from the stirred tank reactor to the plug flow reactor after connection of the two reactors.

### Preculture, batch cultivation and continuous cultivation

A small amount of glycerol stock seed culture was spread onto 2xYT agar plates and incubated at 37°C for 24 h. A single colony was picked to inoculate 500 ml baffled shaking flasks with 50 ml of preculture minimal media. Flasks were then incubated at 37°C on an orbital shaker set to 150 rpm for 16 h. In the next morning 500 µl of biosuspension were transferred to 1000 ml baffled shaking flasks containing 100 ml preculture minimal media and incubated at 37°C on an orbital shaker set to 150 rpm for 8 h. 50 ml of this culture were used to inoculate the bioreactor. Total volume in the bioreactor was 1.6 l after inoculation. Batch fermentation in the bioreactor ensued at 28‐30°C overnight. In the next morning feed and harvest trains were connected and a constant feed/harvest rate at 5.33 ml min^‐1^ corresponding to a dilution rate of 0.2 h^‐1^ established. After 25 h (five volumetric residence times) of STR cultivation a reference sample was taken. The plug‐flow reactor was then connected to the primary reactor via a diaphragm metering pump effectively circulating about one‐quarter of the total fermentation broth from the STR through the PFR and back into the STR. In the following 28 h samples were taken at predefined time points from the STR and the five PFR ports. After 28 h of STR‐PFR cultivation the fermentation was aborted, and the final broth volume measured. This value was used for all volumetric calculations during data analysis.

### Determination of optical density and biomass

In preliminary experiments with identical setup correlation factors of optical density and biomass as cell dry weight (CDW) were determined for *E. coli* MG1655 and *E. coli* SR (Supporting information, Table S1). The resulting correlation factors for converting OD_600nm_ values to g l^‐1^ cell dry weight were 0.324 for *E. coli* MG1655 and 0.321 for *E. coli* SR. In the main cultivations optical density was measured from appropriately diluted broth on a spectrophotometer at 600 nm and converted into biomass concentration.

### Determination of acetic acid, ammonium and glucose concentrations

Five millilitres of biosuspension was directly sampled into a syringe connected to a single‐use 0.45 µm sterile filter and immediately sterile filtered. The clear supernatant was flash frozen in liquid nitrogen and stored at −70°C until analysis. Glucose concentration was determined by D‐Glucose UV‐Test Kit (R‐Biopharm, Darmstadt, Germany) and acetic acid concentration by Acetic acid UV‐Test Kit (R‐Biopharm, Darmstadt, Germany). Ammonium concentration was determined by Ammonium cuvette test LCK 304 (Hach Lange, Düsseldorf, Germany). At the end of the cultivation feed samples were taken and processed identically.

### Analysis of total carbon, inorganic carbon and biomass composition

For total carbon and inorganic carbon analysis 0.5 ml biosuspension sample were mixed with 50 µl of 5 M KOH to prevent loss of dissolved carbonate. The suspension was then diluted 1:20 with demineralized water and stored at 4°C until analysis. Analysis was performed with a multi N/C 2100 S composition analyzer (Analytik Jena, Jena, Germany) to yield the total concentration of carbon and inorganic carbon in the fermenter effluent stream. At the end of the cultivation feed samples were taken and processed identically.

To determine biomass composition 1.0 ml of biosuspension was centrifuged at 4°C and 14 000 rpm (20817 g) for 3 min. The supernatant was discarded, the pellet resuspended in 1.0 ml of freshly prepared 0.9% NaCl solution and centrifuged again. The pellet was resuspended in 5 ml 0.9% NaCl, flash frozen in liquid nitrogen and stored at −70°C until analysis. Analysis was performed with a multi N/C 2100 S composition analyzer (Analytik Jena, Jena, Germany) and the carbon content of the biomass calculated from these values.

### Measurement of ppGpp

Two millilitres of of biosuspension was sampled directly into 0.5 ml of precooled (< −20°C) quenching solution and incubated at 6°C on a shaker for 15 min. Quenching solution consisted of 80 µM EDTA dissolved in 35% (V/V) perchloric acid. 500 µl 1M K_2_HPO4 was added and the sample briefly vortexed. 550 µl 5 M KOH was added and the sample vortexed again. To remove precipitating potassium perchlorate samples were then centrifuged at 4°C and 7830 rpm (7197 g) for 5 min. 1.5 ml of supernatant was carefully transferred to new tubes, flash frozen in liquid nitrogen and stored at −70°C. Prior to analysis samples were thawed and their pH adjusted to 6.95 – 7.05 with 5 M KOH or 35% (V/V) perchloric acid. Samples were centrifuged again to remove all potassium perchlorate precipitate. HPLC analysis was carried out as described previously (Löffler *et al*., 2016). If necessary, quantification was conducted by ppGpp standard addition (TriLink, San Diego, CA, USA). Samples from one time‐point were analysed directly in sequence and the data normalized to the sample drawn from the STR to eliminate differences caused by column aging.

### Transcriptome analysis

0.5 ml broth was sampled from the bioreactor and directly flash‐frozen in liquid nitrogen. Frozen broth was then stored at −70°C until the day of RNA isolation. Total RNA was isolated using RNeasy Mini Kit (Qiagen, Hilden, Germany) according to the manufacturer’s instructions. Isolated RNA was DNAse treated and shipped to commercial sequencing partner GENEWIZ® on dry ice. Samples were treated for rRNA depletion, sequencing libraries prepared and Illumina HiSeq 2x150 bp sequencing performed. Raw FASTQ files were obtained for bioinformatic analysis. Trimmomatic v. 0.32 (Bolger *et al*., 2014) was used to remove adapters and low‐quality reads (<Q20) checked by fastqc reports. Genes were aligned to the NCBI *E. coli* K‐12 MG1655 reference genome (RefSeq: NC_000913.3) using the RNA‐sequencing aligner Bowtie2 v. 2.3.2.2 (Langmead and Salzberg, 2012). On average the mapping of the reads covered 96.2%. Aligned reads were counted for each gene based on the corresponding annotation available from the NCBI database for the chosen reference sequence applying HTseq‐count v. 0.6.1 in the union mode (Anders *et al*., 2015). On average 86.4 % of the sequenced reads could be assigned uniquely to annotated features. Sequencing depth was around 27 million reads per sample on average with a mean quality phred score of 37.63.

Differential gene expression analysis was performed with the R‐package DeSeq2 v. 1.26.0 (Love *et al*., 2014) available from Bioconductor (Gentleman *et al*., 2004). Prior to statistical analysis, all residual non‐protein encoding RNA molecules (tRNA, rRNA and sRNA) were removed from the HTseq‐derived raw count data and a non‐specific filter was applied to remove low coverage genes with fewer than two counts per million (54 reads on average). All filtering steps caused deviations from the raw data of less than 6 %. Samples were grouped by replicates and an experimental design was chosen that used sample time and location (STR or PFR port 5) as a combined environmental factor. To normalize read counts for the comparison of sequencing depth and RNA composition, DESeq2 uses the median of ratios method to derive a scaling factor. Dividing the original read counts by the scaling factor generated normalized count values. No outliers were observed in the two biological replicates using Pearson correlation. Resulting p‐values were adjusted for multiple testing according to control the false discovery rate (FDR) (Benjamini and Hochberg, 1995). Genes were identified as significantly differentially expressed by applying FDR adjusted *P*‐values < 0.01 and a log_2_ fold change ≥ |1|.

A principal component analysis was used to display the sample to sample distances calculated within the DESeq2 package (negative binomial distribution model). Principal component analysis was performed using plotPCA.san available on Github (https://gist.github.com/sansense/3399064897f1252d31b23ea5178c033c).

Gene set enrichment and overrepresentation analysis of up‐ and downregulated genes were performed using the Bioconductors‘s R‐package GAGE v. 2.36.0 (Luo *et al*., 2009). GAGE tests whether the mean fold‐change of a gene subset is significantly different from the background using a two‐tailed t‐test. Genes were selected as significantly different with an FDR adjusted *P*‐value < 0.01 (Benjamini and Hochberg, 1995). Functional annotation were derived from the Cluster of Orthologous Groups (COG) database (Tatusov *et al*., 2003), the experimental sigma factor‐gene interaction dataset from RegulonDB v. 10.6.3 (Santos‐Zavaleta *et al*., 2019) and the Gene Ontology (GO) Groups database with the function go.gsets from GAGE (Luo *et al*., 2009). Furthermore, Venn diagrams were used to identify significant genes shared by both strains and differences in gene expression regulation (Chen and Boutros, 2011).

The RNA sequencing data derived from periodic ammonia starvation experiments have been deposited in NCBI’s Gene Expression Omnibus (GEO) and are accessible through GEO series accession number GSE158198 (Edgar *et al*., 2002). Raw counts and processed data can be found in the Supporting information. Data analysis was performed using the free statistical computing environment R v. 3.6.2.

## Conflict of interest

The authors declare that they have no conflicts of interest.

## Author contributions

Prof. Dr.‐Ing. Ralf Takors advised the study during the entire investigation. Martin Ziegler performed the experiments, and Julia Zieringer conducted the transcriptomic analysis. Evaluation and writing of the manuscript were equally accomplished by Martin Ziegler and Julia Zieringer.

## Supporting information


**Table S1**. Long term changes STR 5min vs STR 0h.
**Table S2**. Long term changes STR 28h vs STR 0h.
**Table S3**. Short term changes PFR28h vs STR28h.
**Table S4**. Short term changes PFR5min vs STR5min.

**Appendix S1**. Supplementary Information.
