# Supplementary material for: Transcriptional profiling of the stringent response mutant strain E. coli SR reveals enhanced robustness to large‐scale conditions
Source: Microb Biotechnol. 2020 Dec 26;14(3):993–1010. doi: 10.1111/1751-7915.13738 (PMC8085953; doi:10.1111/1751-7915.13738)
Supplement: Supplementary file 3 [file MBT2-14-993-s003.pdf]

Supplementary Information

A: Determination of conversion factors from OD 600 nm to cell dry weight concentration

In preliminary experiments, optical density of appropriately diluted fermentation broth from the primary reactor was measured in triplicates at 600 nm on a spectrophotometer (Amersham Biosciences/GE Healthcare, Amersham, United Kingdom). In parallel, quadruplicate determination of biomass concentration was conducted. 5 ml of broth were centrifuged in weighted glass tubes at 2500 g and 4 °C for 7.5 min. Supernatant was immediately decanted and the pellet washed by resuspending in 5 ml of freshly prepared 150 mM NH_4HCO_3. The suspension was centrifuged again and the washing repeated once. After a final centrifugation the pellet was dried at 105 °C for at least two days and the new weight of the glass tubes measured. The ratio CDW [g/l] / OD 600 nm was calculated and averaged over all samples to yield conversion factors for each strain. Conversion factors were 0.324 for *E. coli* MG1655 and 0.321 for *E. coli* SR and used in all subsequent experiments to convert OD 600 nm measurements to cell dry weight concentration [g/l].

Table S1: OD 600 nm and CDW [g/l] data from preliminary experiments.

***E. coli* MG1655**			***E. coli* SR**		
**OD 600 nm**	**CDW [g/l]**	**Ratio CDW / OD**	**OD 600 nm**	**CDW [g/l]**	**Ratio CDW / OD**
Experiment 1			Experiment 1		
8.600	2.545	0.296	8.600	2.975	0.346
8.463	2.875	0.340	8.567	2.925	0.341
8.200	2.550	0.311	8.217	2.815	0.343
8.250	2.555	0.310	8.250	2.865	0.347
Experiment 2			Experiment 2		
8.800	2.720	0.309	9.300	2.665	0.287
8.567	3.140	0.367	9.467	2.790	0.295
8.317	2.925	0.352	9.000	2.715	0.302
8.600	2.685	0.312	9.133	2.790	0.305
**Conversion Factor**		0.324			0.321

B: Transcriptomic analysis of long term response STR vs STR 0h

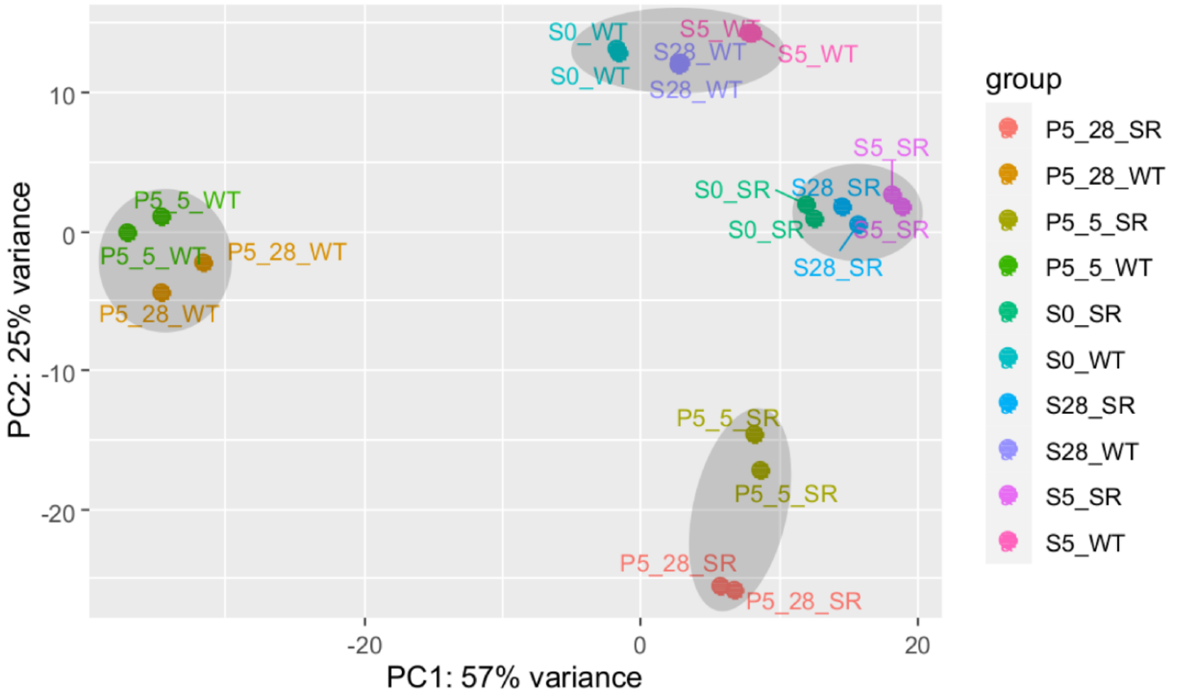

Fig. A1: Principal component analysis of transcript data of *E. coli* MG1655 (WT) and *E. coli* SR obtained from STR (S) and PFR (port 5, P5) at three process time points (0 h, 5 min, and 28 h). Covered measurement variance of each principal component (PC) is indicated. Ellipses cluster samples of STR and PFR. PC1 accounts for 'sample port location', PC2 for 'process time'.

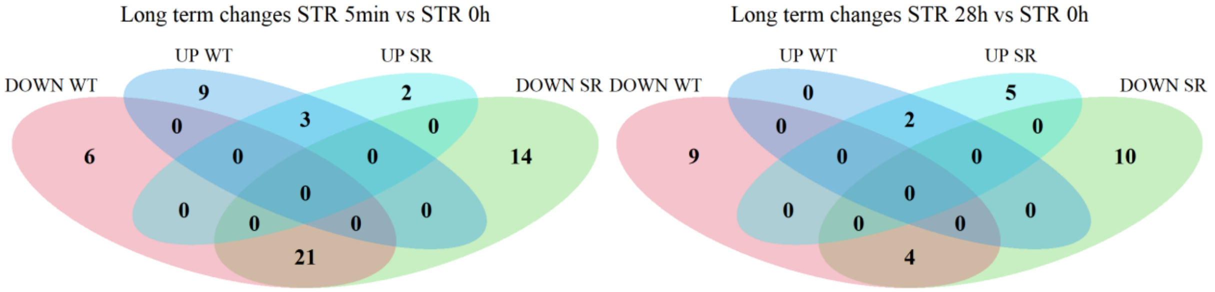

Fig. A2: Venn diagrams representing (overlapping) sets of differentially expressed genes derived from repeated ammonia shortage STR-PFR experiments. Long-term response observed for the following comparisons at PFR sample port long term changes (STR vs. STR 0h) conducted after 5 min of process time (left) and 28 h (right). The number of significantly up- and downregulated genes in each set is indicated by numbers. DEGs were defined as having an FDR < 0.01 and $\log_2$fold change > |1|. Complete gene lists of the Venn diagrams are available in the supplementary information table S1 and S2.

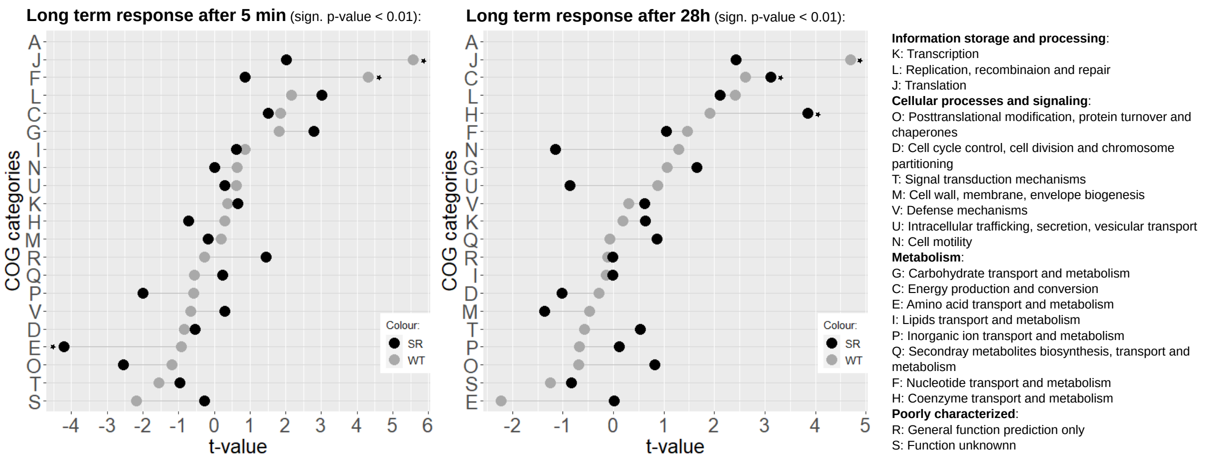

Fig. A3: Left: COG categories long term response 5min STR vs STR 0h WT vs SR; Right: COG categories long term response 28h STR vs STR 0h WT vs SR. Sign. categories are indicated with an asterix.

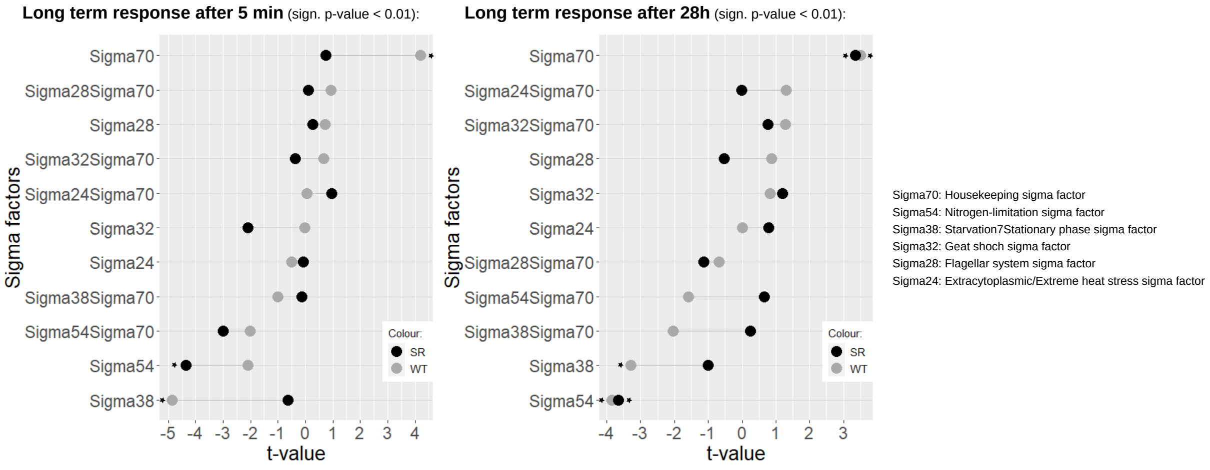

Fig. A4: Left: Sigma factors long term response 5min STR vs STR 0h WT vs SR. Right: Sigma factors long term response 28h STR vs STR 0h WT vs SR. Sign. categories are indicated with an asterix. Sigma70: Housekeeping; Sigma54: Nitrogen-limitation; Sigma38: Starvation/Stationary phase; Sigma 32: Heat shock; Sigma28: Flagellar system; Sigma24: Extracytoplasmic/Extreme heat stress

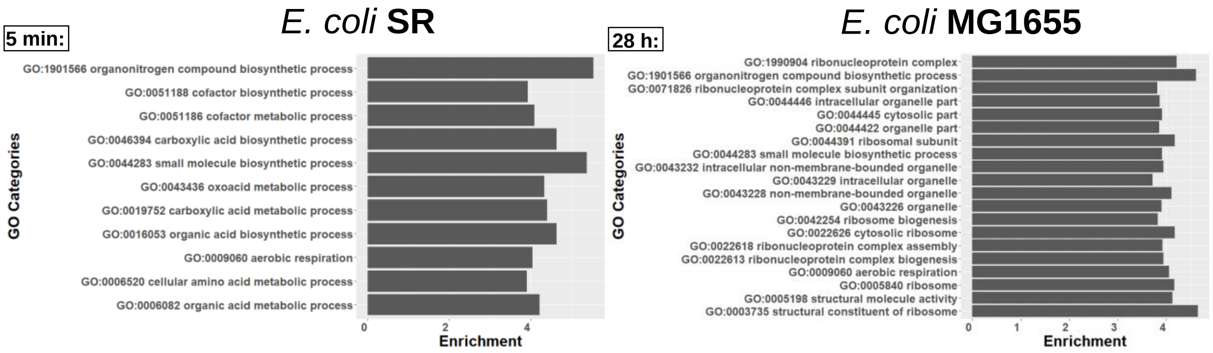

Fig. A5: Left: GO categories long term response 28h STR vs STR0 SR. Top 20 significantly upregulated. No sign. down regulated categories. Right: GO categories long term response 28h STR vs STR0 WT. Top 20 significantly upregulated. No sign. down regulated categories.

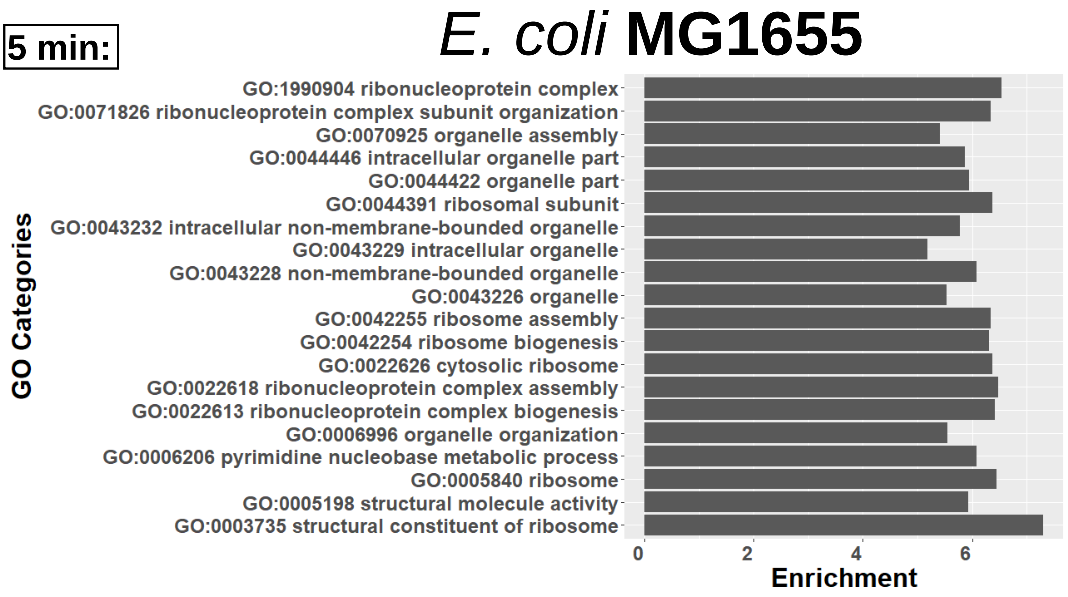

Fig. A6: GO categories long term response 5min STR vs STR0 WT. Top 20 significantly upregulated. No sign. down regulated categories.

C: NtrC-mediated operons

Table S2: NtrC-mediated operons (Brown *et al*., 2014); *E. coli* MG1655

**Operons**	**Log_2FC [a]**		**Function/Pathway**
	**5min short**	**28h long**	
glnK-amtB	3.81	4.09	GlnK - Nitrogen regulatory protein, AmtB – ammonia transport
gltIJKL	1.14	0.94	Glutamate / aspartate ABC transport
dicC			DNA-binding transcriptional repressor
glnHPQ	3.41	3.37	Glutamine ABC transport
yeaGH	2.98	2.69	YeaG is a serine protein kinase
ycdMLKJIHG	4.01	4.05	Pyrimidine degradation
flgMN	0.29	0.84	Regualtion of flagellar synthesis and flagellar biosynthesis protein
ddpXABCDF	4.25	4.49	D-ala-D-ala dipeptide transport and dipeptidase
astCADBE	4.84	4.81	Arginine catabolic patway
fliC	0.46	0.41	Flagellar biosynthesis component
Nac-cbl	4.14	3.94	Nitrogen limitation response-adpater for sigma 70 dependent genes
hisJQMP	1.24	1.64	Histidine ABC transporter
argT	3.32	3.44	Lysine/arginine/ornithine ABC transporter
relA	1.29	1.60	GDP pyrophosphokinase involved in stringent reponse
ssrS	-	-	6S RNA involved stationary phase reulation of transcription
ygjG	3.03	2.79	Putrescine degradative pathway
yhdWXYZ	2.75	2.36	Polar amino acid transport
glnALG	2.57	1.99	Glutamine biosynthesis pathway (ammonia assimilation) and nitrogen regualtion
soxR	0.30	0.27	SoxR transcriptional regualtor
yjcZ-proP	-0.39	-0.26	Hypothetical protein (YjcZ) + symporter (proP)
potFGHI	0.92	0.82	Putrescine transport

[a] Underlining indicates significant differential expression. Logarithmic ratios are always given for the first gene in the transcription unit.

Table S3: NtrC-mediated operons (Brown *et al.*, 2014); *E. coli* SR

Operons	Log_2FC [a]		Function/Pathway
	5min short	28h long	
glnK-amtB	4.05	2.67	GlnK - Nitrogen regulatory protein, AmtB – ammonia transport
gltIJKL	0.25	-0.29	Glutamate / aspartate ABC transport
dicC	-0.09	-0.25	DNA-binding transcriptional repressor
glnHPQ	2.82	2.45	Glutamine ABC transport
yeaGH	1.30	0.89	YeaG is a serine protein kinase
ycdMLKJIHG	3.18	2.83	Pyrimidine degradation
flgMN	0.63	0.73	Regualtion of flagellar synthesis and flagellar biosynthesis protein
ddpXABCDF	4.71	3.12	D-ala-D-ala dipeptide transport and dipeptidase
astCADBE	4.11	3.93	Arginine catabolic patway
fliC	0.05	0.17	Flagellar biosynthesis component
Nac-cbl	3.13	1.42	Nitrogen limitation response-adpater for sigma 70 dependent genes
hisJQMP	0.77	0.59	Histidine ABC transporter
argT	2.51	2.01	Lysine/arginine/ornithine ABC transporter
relA	1.35	1.79	GDP pyrophosphokinase involved in stringent reponse
ssrS	-	-	6S RNA involved stationary phase reulation of transcription
ygjG	2.06	2.16	Putrescine degradative pathway
yhdWXYZ	2.00	1.21	Polar amino acid transport
glnALG	2.64	-0.11	Glutamine biosynthesis pathway (ammonia assimilation) and nitrogen regualtion
soxR	0.34	1.12	SoxR transcriptional regualtor
yjcZ-proP	-0.77	-0.39	Hypothetical protein (YjcZ) + symporter (proP)
potFGHI	0.44	-1.91	Putrescine transport

[a] Underlining indicates significant differential expression. Logarithmic ratios are always given for the first gene in the transcription unit.

D: Motility Assay of E. coli MG1655

A 5 ml overnight preculture of *E. coli* MG1655 in 2xTY medium was incubated at 37 °C and 130 rpm. On the next day, a tube containing motility agar (10 g/l tryptone, 3 g/l yeast extract, 5 g/l NaCl, 0.5 g/l triphenyl-tetrazolium chloride and 0.4 % agar-agar) was inoculated by dipping a thin sterile steel wire into the liquid preculture and stabbing the wire into the motility agar. The culture was incubated without shaking at 37 °C overnight. The spreading of cells as indicated by the expanse of red color from reduction of triphenyl-tetrazolium chloride to triphenylformazan was recorded on the next morning. The formation of a cloudy deep red colored zone indicates strong motility and cell viability.

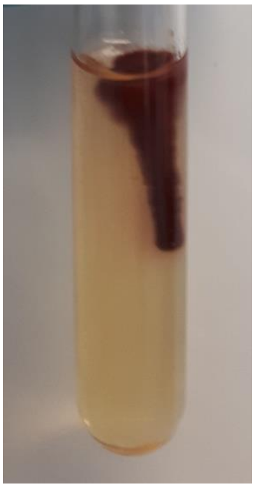

Fig. A7: Motility Assay of E. coli MG1655.

E: Glucose-specific phosphotransferase system components

Table S6: Logarithmic expression ratio and percentage change of mean expression levels from three glucose-specific phosphotransferase system components identified in *E. coli* MG1655.

Gene	Log_2FC^a of *E. coli* SR vs MG1655 (S/S)		Relative Mean Expression Level *E. coli* SR to *E. coli* MG1655	
	**5 min**	**28 h**	**5 min**	**28 h**
ptsG	-0.35	0.67	+ 21.58 %	+ 58.98 %
ptsH	0.57	0.68	+ 48.01 %	+ 60.32 %
ptsI	0.31	0.23	+ 23.82 %	+ 17.28 %

[a] Underlining indicates significant differential expression.
